# Machine learning and word sense disambiguation in the biomedical domain: design and evaluation issues

**DOI:** 10.1186/1471-2105-7-334

**Published:** 2006-07-05

**Authors:** Hua Xu, Marianthi Markatou, Rositsa Dimova, Hongfang Liu, Carol Friedman

**Affiliations:** 1 Department of Biomedical Informatics, Columbia University, 622 168^th ^St, New York City, New York, USA; 2 Department of Biostatistics, Columbia University, 722 168^th ^St, New York City, New York, USA; 3 Department of Biostatistics, Bioinformatics and Biomathematics, Georgetown University Medical Center, 4000 Reservoir Rd, Washington DC, USA

## Abstract

**Background:**

Word sense disambiguation (WSD) is critical in the biomedical domain for improving the precision of natural language processing (NLP), text mining, and information retrieval systems because ambiguous words negatively impact accurate access to literature containing biomolecular entities, such as genes, proteins, cells, diseases, and other important entities. Automated techniques have been developed that address the WSD problem for a number of text processing situations, but the problem is still a challenging one. Supervised WSD machine learning (ML) methods have been applied in the biomedical domain and have shown promising results, but the results typically incorporate a number of confounding factors, and it is problematic to truly understand the effectiveness and generalizability of the methods because these factors interact with each other and affect the final results. Thus, there is a need to explicitly address the factors and to systematically quantify their effects on performance.

**Results:**

Experiments were designed to measure the effect of "sample size" (i.e. size of the datasets), "sense distribution" (i.e. the distribution of the different meanings of the ambiguous word) and "degree of difficulty" (i.e. the measure of the distances between the meanings of the senses of an ambiguous word) on the performance of WSD classifiers. Support Vector Machine (SVM) classifiers were applied to an automatically generated data set containing four ambiguous biomedical abbreviations: *BPD*, *BSA*, *PCA*, and *RSV*, which were chosen because of varying degrees of differences in their respective senses. Results showed that: 1) increasing the sample size generally reduced the error rate, but this was limited mainly to well-separated senses (i.e. cases where the distances between the senses were large); in difficult cases an unusually large increase in sample size was needed to increase performance slightly, which was impractical, 2) the sense distribution did not have an effect on performance when the senses were separable, 3) when there was a majority sense of over 90%, the WSD classifier was not better than use of the simple majority sense, 4) error rates were proportional to the similarity of senses, and 5) there was no statistical difference between results when using a 5-fold or 10-fold cross-validation method. Other issues that impact performance are also enumerated.

**Conclusion:**

Several different independent aspects affect performance when using ML techniques for WSD. We found that combining them into one single result obscures understanding of the underlying methods. Although we studied only four abbreviations, we utilized a well-established statistical method that guarantees the results are likely to be generalizable for abbreviations with similar characteristics. The results of our experiments show that in order to understand the performance of these ML methods it is critical that papers report on the baseline performance, the distribution and sample size of the senses in the datasets, and the standard deviation or confidence intervals. In addition, papers should also characterize the difficulty of the WSD task, the WSD situations addressed and not addressed, as well as the ML methods and features used. This should lead to an improved understanding of the generalizablility and the limitations of the methodology.

## Background

The use of large-scale experimental and information technologies has dramatically increased the pace of production of biomedical findings, and the number of scientific articles has grown rapidly as well, which makes it impossible for human to retrieve or keep up to date with all the related information from the literature. During the last few years, there has been a surge of interest in information extraction and text mining of the biomedical literature [1,2]. When mining the biomedical literature, a big challenge is the problem of ambiguity inherent in natural language because one textual term may have several different meanings or senses (homonymy). A number of natural language processing systems in the biomedical domain reported decreased precision due to the ambiguity problem [3,4]. Weeber [5] found that in order to replicate Swanson's literature-based discovery of the involvement of magnesium deficiency in migraine, it was important to resolve the ambiguity of an abbreviation *mg*, which can denote either *magnesium *or *milligram*.

WSD is very critical for the biomedical text processing community but also very difficult because of the rapid growth of new words and new senses due to a large increase in discovery of biomedical entities. In 2000, the UMLS Metathesaurus [6], a comprehensive resource that specifies and categorizes biomedical concepts, contained 9,416 ambiguous terms, and in 2004, the number increased to 21,295, an increase of 126% within 4 years [7]. More importantly, this figure does not include the many terms associated with gene or gene products, and therefore the amount of ambiguity is likely to be much larger. Studies associated with gene names have shown that the ambiguity problem is complicated because a gene term: 1) may refer to a gene or another type of biomedical term [8], or to a general English word [9]; 2) may be used to denote an RNA, a protein, or a gene [10]; or 3) may be highly ambiguous across multiple species [11]. If each ambiguous gene symbol in an article were accompanied by its corresponding long form, the disambiguation task would be much easier. However, Schuemie [12] analyzed 3,902 biomedical full-text articles and found that only 30% of the gene symbols in the abstracts were accompanied by their corresponding full names, and only 18% of the gene symbols in the full text were accompanied by their gene names. Schijvenaars [13] showed that 33% of the human genes in their thesaurus were affected by homonymy. Chen [11] found that 85.1% of mouse genes were ambiguous with other gene names and 233% additional 'gene' instances were retrieved when gene names that were also English words were included when processing the literature.

To demonstrate the extent of the ambiguity problem in MEDLINE we searched MEDLINE abstracts to determine how many abstracts contained gene symbols that were ambiguous with general English words or biomedical terms. Using data from Entrez Gene[14], the gene-specific database at the National Center for Biotechnology Information (NCBI), we formed two ambiguous word lists for the mouse organism: a gene-English list (containing mouse gene symbols ambiguous with general English words) and a gene-UMLS list (containing mouse gene symbols ambiguous with biomedical terms from UMLS). Then we searched 82,922 abstracts that are known to be related to mouse genes (based on *gene2pubmed *file from Entrez Gene, downloaded on 1/2006) to determine the number of abstracts that contained at least one ambiguous word in each of the above two lists respectively, so that we could determine the percent of abstracts that contained a word that was ambiguous with an English word or with a UMLS term respectively. We repeated the same procedure for the fly and yeast organisms as well. Results showed that for the mouse organism alone, 99.7% (82694/82922) of the abstracts were affected by an ambiguity between a gene symbol and a general English word, and 99.8% (82736/82922) were affected by an ambiguity between a gene symbol and a UMLS term. For the fly organism, both numbers were also over 99%, while the number was much less for the yeast organism: 4.6% and 3.1% respectively. To demonstrate that the ambiguity problem is not limited to a small set of words, we systematically removed ambiguous words with a frequency (ratio between the number of abstracts containing the word and the total number of abstracts searched) higher than a threshold and re-calculated the percentage of abstracts that contained the remaining ambiguous words. In order to reduce the percent of abstracts with ambiguity from gene-English and gene-UMLS to a relative low level (7.2% and 13.4% respectively), ambiguous words with frequencies higher than 0.05% would have to be removed, which covered 30.0% (319 out of 1,065 words) and 30.8% (636 out of 2064 words) of all the ambiguous words in the two lists respectively. The same study, which was also performed for the Fly organism, showed similar results, but with slightly higher ambiguity rates. This study shows that the ambiguity among gene symbols, English words and other biomedical terms is extensive and the distribution of ambiguity is very sparse. This study therefore demonstrates that word sense disambiguation is critical for biomedical text mining and retrieval tasks because ambiguous words have a substantial affect on performance. For the details of the ambiguity study, please refer to the sub-section "Gene Ambiguity for mining MEDLINE" in the Methods section.

Research in automated WSD can be traced back to the 1950s [15]. A number of WSD methods have been addressed for the general English domain. More recently, supervised machine learning (ML) technologies have received considerable attention and have shown promising results [16-18]. Bruce [19] applied a Bayesian algorithm and chose features based on their "informative" nature. They tested their methods on the *interest *corpus, which is a corpus consisting of 6 different senses for the word *interest*, and achieved a precision of 79%. Lee [20] evaluated a variety of knowledge sources (including the parts-of-speech of neighbouring words, single words in the surrounding context, local collocations, and syntactic relations) and supervised learning algorithms (including Support Vector Machines (SVM), Naive Bayes, AdaBoost, and decision tree algorithms) for WSD on the SENSEVAL-1 and SENSEVAL-2 [21] data. Using all of the knowledge sources, the SVM method achieved the highest accuracy rate of 65.4%. Mohammad [22] studied the contribution of lexical features and syntactic features to WSD, and results showed that simple lexical features (words in context and collocation) used in conjunction with part of speech information achieved better results (an accuracy of 66.7% on Senseval-2 set) than other feature combinations.

Another type of WSD approach uses established knowledge from curated terminology systems [23,24]. In the biomedical domain, Schijvenaars [13] developed a simple thesaurus-based algorithm to disambiguate human gene symbols using training data from PubMed abstracts and annotations from the Online Mendelian Inheritance in Man(OMIM)[25]. The system achieved an accuracy rate of 92.7% on an automatically generated testing set. Schijvenaars's study described an effective method for gene disambiguation, but the evaluation results were limited to certain conditions. The automatically generated testing set contained human genes symbols that appeared as long-form and short-form pairs (e.g. prostate specific antigen (PSA)) in articles, where at least 6 articles were determined to be associated with each gene sense. However, in situations where the gene symbol in the paper is ambiguous with a common English word or other type of biomedical word, which is not an abbreviation (i.e. the long form-short form pair is not applicable), the performance of the method is not known: a complete non-abbreviated word may have different characteristics in the text than an abbreviation. For example, this method may not be appropriate for testing a word such as "blind", which is not an abbreviation, but refers to both a gene and a general English word. An additional issue is that this study limited the disambiguation of gene symbols to gene senses and one other category called "non-gene sense", but the actual sense in this category was not resolved. This could be critical for NLP systems accessing phenotypic or disease-related information. An additional limitation of a knowledge-based method is that terms associated with phenotypic senses or general English senses may have little reliable background knowledge available. Therefore, this type of method may not be applicable and ML approaches may be useful. Recently, Humphrey[26] proposed another type of statistical-based method to resolve the ambiguity problem within the UMLS Metathesaurus. They used a Journal Descriptor Indexing (JDI) method, which is ultimately based on statistical associations between words in a training set of MEDLNE citations and a small set of journal descriptors assumed to be inherited by the citations. On a testing set with 45 ambiguous strings from NLM's WSD Test Collection, the overall average precision for the highest-scoring JDI version was 0.7873 compared to 0.2492 for the baseline method.

Supervised ML methods have also been applied to WSD in the biomedical domain. Hatzivassiloglou[10] developed a disambiguation system to determine the class of a known biomedical named entity by choosing one of three pre-defined senses: gene, RNA, protein. He investigated the contribution of different features: positional information of surrounding words, capitalization information, stop-words and similarly distributed word removal, and stemming, and obtained accuracy rates up to 85% with optimised feature combination. Ginter [27] introduced a new family of classifiers, which were based on an ordering and weighing of the feature vectors obtained from word counts and word co-occurrence in the text. This method was used to determine whether a term was a gene versus a protein and achieved 86% accuracy. Podowski [28] built a two-step classification system to disambiguate gene symbols: the first classifier determined whether the word was a gene versus a non-gene, and the other determined the appropriate gene for a symbol classified as a gene by the first classifier. They reported an F-measure of over 0.7 for genes with sufficient number of known document references. Liu [29] investigated the effect of window size and claimed that biomedical ambiguous words needed a larger window size than general English ambiguous words. In Liu's [8] paper, the gold standard data set was automatically constructed utilizing the fact that authors sometimes define abbreviations when they are first introduced in documents using parenthesized expressions [e.g. *Androgen therapy prolongs complete remission in acute myeloblastic leukemia (AML)*] and that the same abbreviation had the same sense within a document. The training data set was automatically annotated using unambiguous synonyms, and for some senses, there were limited samples (e.g. *PCA *with the sense "posterior communicating artery" consisted of only 5 abstracts) for certain datasets. In this study, we used 4 abbreviations from Liu's abbreviation list. However, we used a different method to collect the datasets because we wanted to control the sample sizes of the senses for our experiments. Leroy [30] tried to reduce the training sample size by supplying external knowledge from the UMLS for supervised machine learning algorithms, but the results were not promising. Gaudan [31] developed an algorithm based on use of SVMs to resolve abbreviations in Medline and claimed a precision of 98.9% and a recall of 98.2% on their testing set. In their study, rare senses (senses appearing in less than 40 documents) were excluded from the testing set. This makes the disambiguation task easier because it reduces the problem of sparse senses. In addition, the training set was created based on long-form and short-form pairs, where ambiguous words not having long-forms were not tested. There is a good review of current research of WSD in biomedical domain by Schuemie [32].

Most of the above papers reporting on the use of ML for WSD follow a similar pattern. A set of ambiguous words is selected, a corpus for each word is collected, and the different senses within the corpus are annotated (automatically or manually). A feature vector is then formed based on the context of the ambiguous word, a supervised machine-learning algorithm is used on a portion of the corpus to train a classifier for the word, and the classifier is tested on the remaining corpus. The main variations are usually in the selection of features and choice of machine-learning algorithms. Experiments are usually performed on a fixed amount of documents (i.e. 1,000 abstracts) per an ambiguous word, where the entire set consists of all the senses, and the sense distribution is generally uneven. Results (usually error rate or accuracy) are reported and an analysis of a few issues is often described, but the results of different experiments are usually not comparable because multiple confounding issues affect them. These papers are important in that they report on useful methods and provide insights and overall results. However, a deeper and more systematic analysis is needed in order to obtain a better understanding of the different factors affecting the performance of ML methods for WSD. In this paper, we discuss a number of issues explicitly and describe some experiments that simulate a variety of situations where different sense distributions, different sample sizes, different levels of difficulties, and different cross validation methods are studied and the effects are quantified. We subsequently based our assessment of performance on error rates and associated standard errors. Although some issues we have addressed in this paper have been mentioned by other papers, our work differs from related work because we focus on a systematic study of issues affecting performance and quantify their effects in order to further understanding of the components of the error rate, which should lead to an improved and more generalizable methodology. Our method also differs from related work because the sample size for each sense is always fixed, whereas in related work the sample size for the entire corpus is generally fixed but not the sample sizes of the senses.

## Results

Four ambiguous abbreviations: *BPD*, *BSA*, *PCA*, and *RSV*, were used in this study. They were chosen because they were associated with varying degrees of differences between their respective senses. For example, two of the senses of *PCA *studied are very similar whereas two senses of *BSA *are very different. Table 1 lists the detailed information about the abbreviations and their senses, and the Methods section explains the differences in more detail. For each abbreviation, we measured error rates of the SVM classifier under different combinations of sample size, sense distribution, cross validation scheme (5-fold vs. 10-fold), and multi-class SVM algorithms (for BPD only, which has 3 different senses). For details of the testing data set and experimental design, please refer to the Methods section.

Tables 2, 3 and 4 display the results for *BSA*, *PCA *and *RSV*, each of which has two senses. The distribution shown with bold font in column 1 is the estimated distribution of the senses, which is calculated based on the number of retrieved articles for each sense and the number of retrieved articles for all the senses. Column 2 is the number of total samples from all senses. The range of sample size per sense ranges from 10–40, with increments of 10 per sense. Average error rates (Err. Rate) and average standard errors (SE) were reported for each combination of distribution and sample size (see Methods section).

With a distribution of (0.5, 0.5) and 5-fold cross-validation, the error rate for *BSA *dropped from 21.83% at sample size 20 to 3.11% at sample size 120. With the same sample size change, the error rate for *PCA *dropped from 43.00% to only 28.53%. Results for *BPD *are shown in Table 5, which contains the results from three different multi-class SVM algorithms. We used Friedman's test [33] to compare the different multi-class algorithms, and stratified the analysis by probability distribution using sample size (four levels) and multi-class algorithm (three levels) as the two factors in the ANOVA table. The analysis, adjusted appropriately for multiple testing, revealed that only the pair ("one-vs-rest", "one-vs-one") differed and there was no statistically significant difference (at overall level α = 0.1) between "mc-svm" and "one-vs-rest" SVM algorithms. This agrees with work by Rifkin and Klatau [34]. A description of the different multi-class algorithms is provided in the Methods section

**Figure 1:**
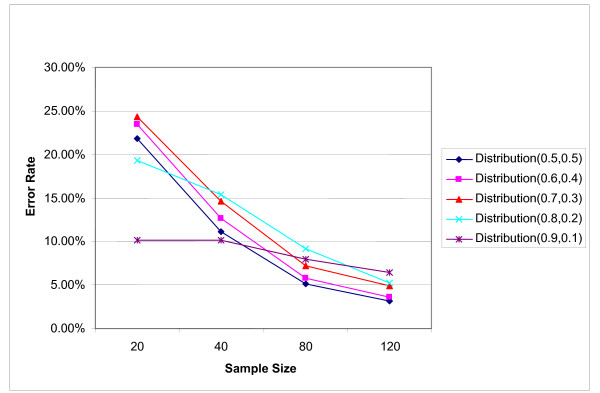
**Error Rate versus Sample Size with different sense distributions of *BSA *data set**. This figure shows the plots of "error rate" versus "sample size" with different sense distributions of *BSA *data set (case where the 2 ambiguous senses are very different) using 5-fold cross-validation.

**Figure 2:**
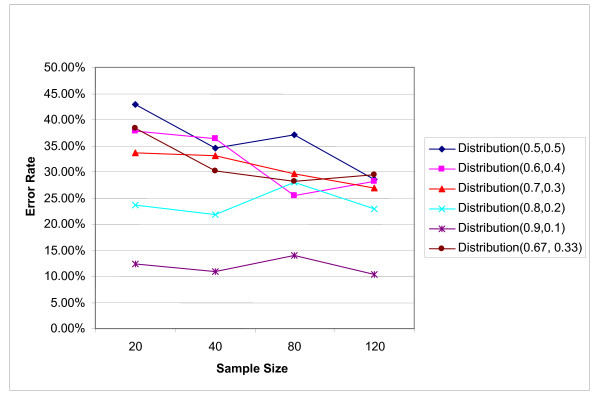
Error Rate versus Sample Size with different sense distributions of *PCA *data set. This figure shows the plots of ''error rate'' versus ''sample size'' with different sense distributions of *PCA *data set (case where the 2 ambiguous senses are very similar) using 5-fold cross validation.

**Figure 3:**
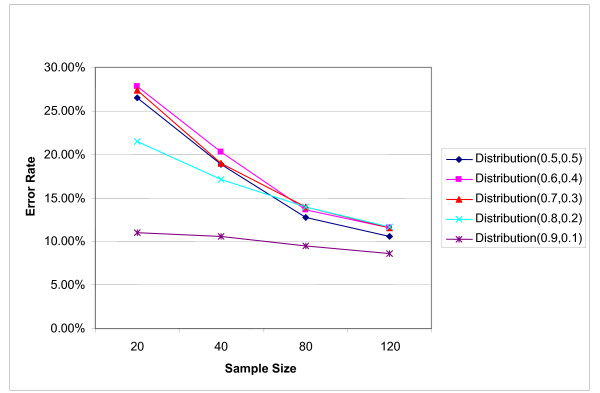
**Error Rate versus Sample Size with different sense distributions of *RSV *data set**. This figure shows the plots of "error rate" versus "sample size" with different sense distributions of *RSV *data set (case where the 2 ambiguous senses both refer to viruses but the viruses are different types of viruses) using 5-fold cross validation.

Figures 1, 2 and 3 show the error rate versus the sample size for each distribution of the *BSA*, *PCA *and *RSV *data sets with 5-fold cross-validation. As the figures indicate, the reduction of the error rate as a function of the sample size is more dramatic for *BSA *than for *PCA*. For *BSA *there is about a four-fold reduction in the error rate when the sample size increases from 20 to 80 for sense distributions (0.5, 0.5), (0.6, 0.4) and (0.7, 0.3), while there is a two-fold reduction for sense distribution (0.8, 0.2) and no reduction for (0.9, 0.1). In contrast, for *RSV*, a two-fold reduction of the error rate was observed for distributions (0.5, 0.5), (0.6, 0.4), (0.7, 0.3) and (0.8, 0.2) for an increase in the sample size from 20 to 80. The distribution (0.9, 0.1) behaved the same as *BSA*.

For *BSA *and *RSV *there was no significant effect of the sense distributions on the error rates for all different sample sizes, but for *PCA *the effect of the sense distribution on the error rate was significant. Multiple comparisons, adjusted for multiple testing, indicated that when the overall significance level is 0.1, the sense distributions (0.5, 0.5) and (0.6, 0.4) impact the error rate. These results show that almost balanced sense distributions and rather large training sample sizes reduce the error rate to approximately half of our best guess, which is using the majority sense.

To address the issue of whether a meaningful reduction in the error rate was achieved by increasing the sample size, we performed further statistical analysis on the results of the *BSA *and *PCA *data set. To test the null hypothesis of no differences in the error rates among the different sample sizes (and overall probability distribution) for the *BSA *and *PCA *abbreviations, we used Friedman's test. Then we performed sub-analysis using the sign-test (see Methods section for details). The results are summarized as follows and they apply to both 5-fold and 10-fold cross-validation schemes. When the senses are well separated, any increase in the sample size results in a statistically significant decrease of the error rate. This holds for all sense distributions and it is in agreement with the finding that for *BSA *there was no significant effect of the sense distributions on the error rates for the different sample sizes used. There are, however, differences when the meanings of the senses are not well separated (e.g. *PCA*). As the Friedman's test indicated, the effect of the sense distribution on the error rate is significant. When the sense distribution is (0.5, 0.5) there are statistically significant differences between the pairs of error rates produced under sample size (20 and 120), the sample sizes (40 and 120) and the sample sizes (80 and 120). The differences in the error rates produced under sample sizes (20 and 40) and (20 and 80) are borderline significant (overall level α = 0.05). When the sense distribution is (0.6, 0.4), an increase in the sample size from 20 to 40 and from 80 to 120 does not produce statistically significant differences in the corresponding error rates. For all other sense distributions, an increase in the sample size did not produce a significant reduction in the error rate – that is, there are no statistically significant differences between the error rates. We would like to stress here a limitation of the current study. This is the fact that the experiments were carried out only 30 times: this rather small number of replication of the experiments may have contributed to observing borderline significance.

**Figure 4:**
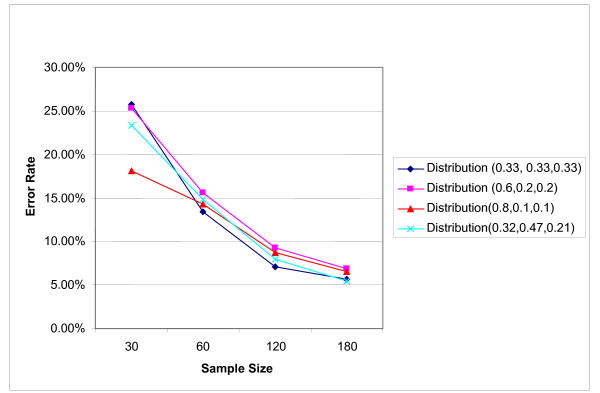
**Error Rate versus Sample Size with different sense distributions of *BPD *data set**. This figure shows the plots of "error rate" versus "sample size" with different sense distributions of *BPD *data set (where there are 3 ambiguous senses that are different) using 5-fold cross validation and "one-vs-rest" algorithm.

**Figure 5:**
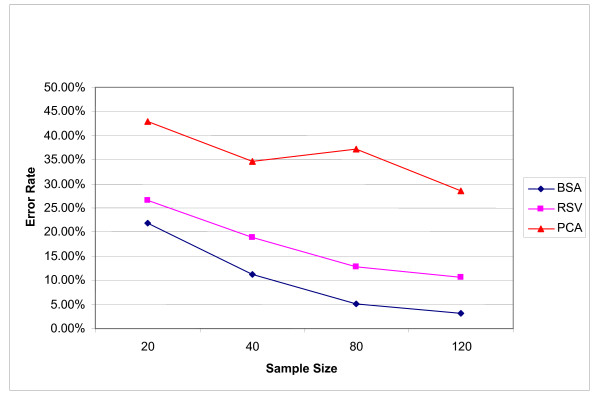
**Error Rate versus Sample Size for *BSA*, *RSV *and *PCA *with sense distribution of "(0.5,0.5)"**. This figure shows the plots of "error rate" versus "sample size" for *BSA*, *RSV *and *PCA *data sets with fixed distribution of "(0.5, 0.5)" using 5-fold cross validation.

Figure 4 shows plots of the error rate versus sample size for each distribution of the *BPD *data set based on the 5-fold cross validation using the "one-vs-rest" algorithm. The plots for the four different sense distributions are very similar and actually agree with results obtained indicating that the effect of the different distributions on the error rate is insignificant. Figure 5 shows error rate versus sample size plots for three different abbreviations (*BSA*, *RSV*, and *PCA*) at the same distribution (0.5, 0.5). It was presented to show the degree of difficulty among different abbreviations. As expected, the error rate had the following order: *BSA *<*RSV *<*PCA*, which indicated that similar meanings were more difficult to classify. Results from 5-fold cross-validation showed no statistical difference with results from 10-fold cross-validation, which indicated 5-fold cross-validation might be used in evaluation in order to save computational power (for a discussion of the relative merits of 5-fold cross-validation vs. 10-fold cross-validation, see[35]).

## Discussion

### Issues and our experiments

"Sample size', "sense distribution" and "degree of difficulty" were three of multiple confounding issues that affect the performance of a WSD classifier. Results from our experiments demonstrated that these three factors were intrinsically connected. Notice that as expected, with any distribution, the error rate generally decreased as the sample size increased. However the observed decrease in error rate was more dramatic in the cases where the different senses were well separated. For example, in *BSA*, the error rate dropped to approximately 5% when the sample size was 80 and the sense distributions were almost balanced, and it was approximately 8% for other distributions with the same size. Notice also the relatively small standard deviations that are associated with those error rates. Moreover, when two senses of a word are very different, then the reduction that is observed in the error rate is meaningful in the sense that it is generally outside the limits of (error rate) ± (1 SE) for increases in the sample size from 20 to 40 to 80. In contrast, when the separation between the two senses is poor (i.e. when the senses of an abbreviation are similar to each other), increasing the sample size does not help much, and a very large increase in size is needed for a small reduction in the error rate. In particular, we notice that when the sense distribution (P1, P2) was very unbalanced (i.e. 0.9, 0.1), then the error rate was almost equal to the minority sense proportion. All these findings indicate that the effectiveness of an increase in the sample size is very dependent on the degree of difficulty. When the degree of difficulty is very high, increasing the sample size will not help much unless an extraordinarily large size is used, which would be very costly.

There are different types of WSD and some are more difficult than others. For example, if two senses are syntactically different, a reliable part of speech tagging method could be effective in resolving the ambiguity. For senses that correspond to the same syntactic category, the similarity of their semantic categories will affect the difficulty of the task (i.e. the *bovine serum albumin *sense of *BSA *is substantially different from the *body surface area *sense). Even for senses within the same semantic class, two close senses will be much more difficult to classify than two unrelated meanings. For example, in *RSV*, both senses (i.e. *respiratory syncytial virus *and *rous sarcoma virus*) are associated with a "virus" concept, but the two concepts are very different types of viruses, and therefore the contexts in which they occur are likely to be different as well. As shown in Figure 5, *PCA*, which has two very close senses, had much higher error rates than *BSA*, which has two unrelated senses. Therefore, when comparing the performance of different WSD systems, data sets with the same degree of difficulty should be used. Resnik [36] stated the importance of the semantic similarity of senses and proposed a method to compute performance, which takes similarity of senses into account. Our study is different because it quantified the effect of similarity of senses, and studied the relation between "similarity of senses" and other issues such as "sample size" and "sense distribution". When considering gene symbol disambiguation, we could categorize the tasks as involving four different types of disambiguation: 1) classifying whether a term is a noun or another syntactic part of speech, such as a verb, in which case the term cannot be a gene; 2) classifying whether a term refers to a gene or a non-gene sense (e.g. a general English word or other biomedical term); 3) classifying which gene a term refers to if it is ambiguous with multiple genes or which non-gene sense a term refers if it is ambiguous with multiple non-gene senses; 4) classifying which product (gene, RNA, Protein) a term refers to if it is known to be a particular gene. Podowski's [28] work covered task types 2 and 3, while Hatzivassiloglou's [10] work addressed task type 4. Many evaluations report their results for a set of words, but the difficulty levels and types of disambiguation task types are not stratified.

To be able to identify whether there are significant differences in the error rates due to different sample sizes and sense distributions while controlling for the abbreviation used, we used Friedman's procedure. Notice that if we stratify by the abbreviation, the mean error rates form a two-way table where the columns correspond to different sample sizes and the rows correspond to different sense distributions. The significance of this methodology is that it provides a comprehensive way to quantify the effects of sample size and sense distribution on the error rate. For *BSA*, *RSV *and *BPD*, we found that the effect of the sense distribution on the error rate was insignificant. For *PCA *this effect was significant. The effect of different sample sizes on the error rate was significant for *BSA*, *RSV*, and *BPD*. For *PCA*, although the effect of sample size on the error rate was significant, this effect was observed only when the sample size was increased from 20 to 120, and for fairly balanced sense distributions such as (0.5, 0.5) and (0.6, 0.4). For those two distributions, an increase from 20 to 80 was also significant. Smaller increases in the sample size had an insignificant effect.

We performed further sub-analysis using non-parametric multiple comparisons to identify the pairs of sample sizes that differ when the abbreviations *BSA *and *RSV *were analyzed. This analysis revealed that in the case of *BSA *the improvements in terms of error rate were statistically significant across distributions as the sample size increased from 20 to 40. For the case of *RSV*, a much more substantial four-fold increase in the sample size was needed in order to observe an appreciable decrease of the error rate. Effects of "sense distribution" have been addressed in other papers [30,37] because it is believed that the performance of a WSD classifier may change if the distribution of the different senses is unbalanced. For example, when there is a majority sense for an ambiguous word, the improvement of a WSD classifier is believed to be very small. Results from our study showed there was a difference only when the distribution was very uneven and the task was difficult. For example, for *PCA*, when the majority sense was over 0.8, the error rate started to decrease and when it was over 0.9, the error rate dramatically decreased so that use of the majority sense was as effective as the ML methods, but with much less cost.

### Other confounding issues of WSD

Other issues in addition to sample size, distribution of senses, and difficulty of the task also affect the performance and subsequent assessment of WSD classifiers, as noted below:

#### • Features used

As often discussed in various papers, different features were evaluated to see their contribution to classifier performance [10,20,29]. From these papers, there was no single combination of features that seemed to be associated with the best results for any type of WSD task. This could also be due to the existence of other confounding factors in the datasets that were used. In our study, we controlled for this factor by using "bag-of-word" features in all experiments, but it would be interesting to see if the performance improves when different feature vectors are used

#### • ML algorithm

Most papers reported that different ML algorithms did not show much difference on performance [29,30]. But some reported that certain classification algorithms were better than others. For example, Mooney [16] did a comparison study among a naïve Bayes classifier, perceptron, decision-tree learner, k-nearest-neighbor classifier, logic-based disjunctive normal form, conjunctive normal form and a decision-list learner, and the results showed that the naïve Bayes and perceptron classifiers performed significantly better than all others. It is still an unclear issue, probably due to the interaction of different combinations of issues. The comparison between different classifiers should be a carefully controlled experiment. The notion that a lower absolute error rate is indicative of the superiority of a classifier is generally flawed because it ignores the possibility that the differences in the different experiments performed are not statistically significant [38]. Statistical tests [39,40] can be used to compare different classifiers.

#### • Baseline reported

It is very important that the baseline of a classification task is reported because it shows how much of an improvement there is using a classifier as compared to the baseline. As shown in our experiments, when there is a majority sense of 0.9 or more, the performance of a WSD classifier may seem high, but that is not due to the classifier. Several papers [29,30] realized this issue and reported results for the baseline. More specifically, they excluded samples with a majority sense larger than a threshold because they realized the contribution of the classifier would not be much for those cases.

#### • Results with confidence intervals

When reporting the results (i.e. error rate), not all papers reported confidence intervals (or a similar metric, such as standard deviations). When comparing the performance of WSD classifiers, those metrics are critical because they indicate whether or not an improvement is statistically significant; if there is a large deviation, there may not actually be an improvement even though one error rate is smaller than the other.

#### • Feasibility

One of the problems of supervised machine learning for WSD is the need for an annotated training (and testing) data set for each ambiguous word, which may require a huge effort. There are two approaches that address this problem: 1) designing an efficient sampling method to lower the cost of manual sense tagging [41], or 2) use of an automated method to generate sense-tagged data [8,42], but this may not always be possible or may inadvertently introduce bias. In our study, we proposed a simple "full-term substitution" method, which is described in more detail in the Methods section, to automatically generate training data, but this is only applicable for abbreviations.

In this study, we used a "full-form substitution" method to automatically generate the data set for the experiments, which is an artificial training set. We compared the estimated sense distribution from our method with that of Liu's method [8] and found they were similar for most of the abbreviations (e.g. *RSV, BPD, BSA*), and that the majority senses based on use of each method were the same. We did not compare the substitution method with other methods for WSD. In addition, we used an SVM classifier for all the experiments. Since the goals of our study did not include the comparison of different algorithms, we do not present related results here. Other studies showed that different ML algorithms had similar performance for WSD tasks [29,30]. Thus, it is likely that our findings are applicable to other ML methods because similar issues have been discussed in the general ML literature [43].

Earlier studies have investigated a number of the issues discussed here in the context of constructing better classifiers. A discussion of some of the issues involved can be found in [43]. Here, we examined these issues in the context of word sense disambiguation. The methodology we used to quantify the impact of various factors on the error rate, and hence on the performance of the WSD classifier, is a well-known, theory-based, statistical methodology. The methodology is easy to apply, it provides a principled way of studying the effects of the different factors on the error rate, and since it is based on a strong theoretical foundation, it guarantees that the results to apply to all abbreviations with similar characteristics. Therefore, although we studied only four abbreviations, the results concerning sample size, sense similarity, and distributions are likely to be generalizable for abbreviations with similar characteristics. The results presented here agree with general results presented in the literature on the performance of classifiers [43-45].

### Future work

To further analyze the effects of "sample size", "sense distribution" and "degree of difficulty" on the error rate, an error decomposition model will be explored. Methods to measure the degree of distances among different senses are also being studied.

## Conclusion

In this paper, we aimed to further an understanding of the different factors affecting the performance of ML techniques for WSD by systematically simulating a variety of situations where different sample size, sense distribution, degree of difficulty, and cross validation methods were used. We evaluated the performance of SVM classifiers for those situations. Results from our experiments showed that: 1) increasing the sample size generally reduced the classifier error rate, but this was limited mainly to well-separated senses (such as senses with different semantic types or senses with the same semantic types but unrelated meanings); in difficult cases an unusually large increase in sample size was needed to increase performance slightly, which was costly and impractical, 2) the sense distribution did not have much effect on classifier performance for cases where the senses were separable, 3) when there was a majority sense of over 90%, choosing the majority sense seemed to be the most effective strategy because the cost was low as was the error rate, 4) the error rate was proportional to the similarity of senses, and 5) there was no statistical difference between results using 5-fold or 10-fold cross-validation. In this paper, we also demonstrated that ambiguity of biomedical entities is a significant problem, which has a substantial impact on text mining and retrieval tasks in the biomedical domain.

ML methods are still needed for WSD, which is critical for increasing the accuracy of biomedical natural language, text mining, and information retrieval systems. ML methods are especially important for those cases that cannot readily be addressed using knowledge-based methods. Therefore it is important that we understand the different elements affecting their performance. In order to improve our understanding of the ML methods, it is critical that in addition to reporting on overall results, papers also report on the baseline performance, the distribution of senses in the datasets, the standard deviation or confidence intervals, the types of ambiguity addressed, and the difficulty of the task as well as the methods and features used.

## Methods

After manually reviewing a set of WSD papers in the biomedical domain, different issues associated with performance were enumerated. For an initial study, we conducted experiments to evaluate the effect of three confounding issues: "sample size", "sense distribution" and "degree of difficulty", and we used an automatically generated data set. A discussion of the results and issues can be found in the Results and Discussion sections.

### Data set for experiments

Four abbreviations were used in the experiments. Table 1 lists the detailed information about the abbreviations and their senses. These abbreviations were originally specified in the ABBR data set [8]. We chose them by considering the different levels of semantic similarity among their senses. *BSA *denotes two senses that have very different meanings, but *PCA *denotes two senses that have very similar meanings; the two senses of *RSV *are both associated with a virus, but the viruses are very different types; finally, *BPD *denotes three very different senses. The original data set for *PCA *contained 6 different senses, but we only used the two that were very similar for our experiments. We used a simple "full-form substitution" method to automatically generate a data set for the experiments described in this paper, and this dataset was partitioned into training and testing sets. To perform the "full-form substitution" for each sense of an abbreviation, PubMed articles published before October 2005 were searched using an exact string match for the full-form of the sense. The full-form in the title or abstract of the article was then replaced with the ambiguous abbreviation, and the appropriate sense was noted separately. Table 1 shows the number of articles that were obtained for the different abbreviations and senses. The estimated sense distribution was calculated from the number of retrieved articles and displayed in the last column. For each sense, we recorded all the retrieved PMIDs, randomly selected 250, and then obtained the corresponding abstracts to form a data pool, from which all the experiments were drawn.

### Feature vector and machine-learning algorithm

For all the experiments in this paper, we used the simple "bag-of-word" method to construct the feature vector. All the words in the title and abstract of the articles were used as features for machine learning and an SVM algorithm was used to generate a classifier. We used a package called "Spider" [46] to perform all the SVM training and testing. For abbreviations with only two senses (*BSA, PCA, RSV*), a binary SVM classifier was used. For *BPD*, which has three different senses, three different multi-class SVM methods [47]: "mc-svm", "one-vs-rest", "one-vs-one", were used. "Mc-svm" implements the algorithm with a decision function which considers all classes at once, while "one-vs-rest" and "one-ve-one" are constructed by combining several binary SVM classifiers. "One-vs-rest", also known as "one-against-all", constructs N binary SVM classifiers for a classification task with N classes. The *i*th binary SVM classifier is trained by considering all instances associated with the *i*th class as positive examples and the others as negative instances. It applies the N classifiers and chooses the one with the highest confidence. "One-vs-one", also known as "one-against-one", constructs N(N-1)/2 binary SVM classifiers where each is trained with data from two classes: one as positive and one as negative. It applies these N(N-1)/2 SVM classifiers and the class assignment is determined by a voting strategy (e.g. the class chose by the maximum number of SVM classifiers wins). The performance was measured using both a 5-fold and a 10-fold cross-validation method.

### Experiments

For abbreviations with two senses (*BSA, PCA, RSV*), we simulated 5 different combinations of sense distribution, which were (0.5, 0.5), (0.6, 0.4), (0.7, 0.3), (0.8, 0.2), (0.9, 0.1), and also used an additional combination, which was the estimated distribution of the senses. For example, a sample testing set with size 20 and sense distribution (0.5, 0.5) means 10 samples in the set are with one sense and the other 10 samples are with the other sense. The estimated sense distribution is listed in the last column of Table 1, which is calculated based on the number of retrieved articles for each sense and the number of retrieved articles for all the senses. For *BSA *and *RSV*, the estimated distributions were the same as one of the 5 simulated distributions, and therefore the experiments used only 5 combinations for those two. For PCA, the estimated distribution was (0.67, 0.33). Four different sample sizes were used (20, 40, 80 and 120), and for each, a proportional sample for each sense was obtained based on the particular distribution. For *BPD*, which has 3 senses, 4 distribution patterns were used: (0.3,0.33,0.33), (0.6, 0.2, 0.2), (0.8, 0.1, 0.1) and (0.32, 0.47, 0.21), where the last one was the estimated distribution. For each distribution pattern, 4 different total sample sizes were used: 30, 60, 120 and 180. Error rates for each combination of sense distribution and sample size were averaged using 30 runs.

### Statistical methodology

To quantify the effects of sample size, sense distribution and difficulty of the task on the error rate, appropriate statistical methods were used. Friedman's test is the non-parametric analogue of a two-way analysis of variance (ANOVA) table. No assumptions are made about the original distribution (e.g. normal vs. other) of the documents. Analysis of variance models are versatile statistical tools for studying the relation between error rates and sense distribution, sample size, and degree of difficulty of a task. These models do not require making assumptions about the nature of the statistical relation, nor do they require that sense distribution, sample size or degree of difficulty to be quantitative variables.

To understand the effects of increased sample size on the error rate, we stratified by the sense distribution and then tested the null hypothesis of no difference between the error rates obtained under the different sample sizes using the sign test. The sign test is a non-parametric test that does not impose any distributional assumptions, such as normality, on the data. It is useful for testing whether one random variable in a pair tends to have larger (smaller or simply different) values than the other random variable in the pair. In our case, the random variables in the pair are the error rates obtained under the different sample sizes used. For each abbreviation, each sense distribution and each cross-validation scheme we have 6 pairs of random variables corresponding to different combinations of the sample size. For each combination of error rates we have a sample of 30 observations. To exemplify, assume the pair consisted of the error rates obtained under sample size 20 and 40. Then the set of observations was comprised of those error rates obtained from the 30 simulation runs. The null hypothesis would be that the median error rate when the sample size is 20 equals the median error rate when the sample size is 40. Because, for each sense distribution, we had 6 such comparisons to make we adjusted for multiple testing by setting the overall significance level α = 0.05 and then divided this by 6 to obtain individual level of 0.0084 (Bonferroni Adjustment).

We computed the standard deviation of the error rate as follows. Recall that for each abbreviation, each sense distribution and each sample size we run the experiment 30 times. Let *p*(*i*) denote the error rate for the *i*th data set, *i *= 1,2,...,30. The error rate was computed using both a 5-fold and a 10-fold cross-validation scheme. Let the size of the training set be denoted by *n*. For example, when the total sample size is 20 and 5-fold cross validation is used the size of the training set is 16, while if the sample size is 80 the size of the training set is 64. For each of the 30 runs we estimated the standard error using the formula:p(i)(1−p(i))/n
 MathType@MTEF@5@5@+=feaafiart1ev1aaatCvAUfKttLearuWrP9MDH5MBPbIqV92AaeXatLxBI9gBaebbnrfifHhDYfgasaacH8akY=wiFfYdH8Gipec8Eeeu0xXdbba9frFj0=OqFfea0dXdd9vqai=hGuQ8kuc9pgc9s8qqaq=dirpe0xb9q8qiLsFr0=vr0=vr0dc8meaabaqaciaacaGaaeqabaqabeGadaaakeaadaGcaaqaaiabdchaWnaabmGabaGaemyAaKgacaGLOaGaayzkaaWaaeWaceaacqaIXaqmcqGHsislcqWGWbaCdaqadiqaaiabdMgaPbGaayjkaiaawMcaaaGaayjkaiaawMcaaiabc+caViabd6gaUbWcbeaaaaa@3B18@. The estimate of the standard error was then obtained by averaging the above values over the 30 runs.

### Gene ambiguity for mining MEDLINE

To determine the extent of the gene ambiguity problem in MEDLINE, we searched MEDLINE abstracts to determine how many abstracts contained gene symbols that were ambiguous with general English words or biomedical terms. We formed a mouse gene symbol list by retrieving all gene symbol/name/synonyms from Entrez Gene[14], the gene-specific database at the NCBI, for the mouse species. Then we compared this gene symbol list with a general English word list (Webster's 2^nd ^international dictionary) and with the UMLS term list (from UMLS Metathesaurus 2005AA, removing all bio-molecular entities with semantic types "Gene or Genome", "Biologically Active Substance", "Amino Acid", "Peptide or Protein", "Enzyme", "Immunologic Factor and Receptor", please see[11] for details) via case-insensitive exact string match. Two ambiguous gene symbol lists were formed as a result of the comparisons: a gene-English list (containing gene symbols ambiguous with general English words) and a gene-UMLS list (containing gene symbols ambiguous with biomedical terms). We also formed a pool of MEDLINE abstracts by collecting all abstracts that were related to mouse genes using *gene2pubmed *file from Entrez Gene (downloaded on 1/2006), which led to 82, 922 abstracts in the pool. We performed a case-insensitive search on each abstract in the pool to determine the number of abstracts that contained at least one word in each of the above two lists respectively, so that we could determine the percent of abstracts that contained a word that was ambiguous with an English word or with a UMLS term respectively. However there is a concern that a very limited set of words may have accounted for the vast majority of ambiguity. Therefore, for each ambiguous word, we calculated its frequency, which is defined as the ratio between the number of abstracts containing the word and the total number of abstracts in the pool. For example, the word "brown" occurred in 399 abstracts therefore had a frequency of 399/82922 = 0.48%. For each threshold, we removed ambiguous words with frequencies higher than that threshold and re-calculated the percentage of abstracts that contained the remaining ambiguous words. Meanwhile, we also recorded the percentage of ambiguous words that were removed from the ambiguous word-list for different thresholds. We removed words with frequencies higher than 10%, 1%, 0.1% and 0.05% from the two lists of the mouse organism. Results showed that the percentages of abstracts containing the remaining ambiguous words were 80.9%, 46.2%, 13.5% and 7.2% respectively for gene-English ambiguity, and 89.8%, 68.6%, 24.0% and 13.4% respectively for gene-UMLS ambiguity. The percentages of ambiguous words that were removed from the list for different thresholds(10%, 1%, 0.1%, 0.05%) were 0.8%(8/1065), 4.8%(51/1065), 20.3%(216/1065), 30%(319/1065) for gene-English ambiguity and 1.0%(20/2064), 3.8%(79/2064), 21.2%(437/2064) and 30.8%(636/2064) for gene-UMLS ambiguity. The same study, which was also performaned for the Fly organism, showed similar results, but with slightly higher ambiguity rates. For a more complete description of this study and the results, please [see additional file 1].

## Authors' contributions

HX carried out data collection, programming, experiments using SVM and drafted the manuscript. RD participated in the statistical analysis of the results. MM and CF conceived of the study, and participated in its design and coordination and helped to draft the manuscript. MM also performed statistical analysis and interpreted the results. HL advised in the design of study. All authors read and approved the final manuscript.

## Supplementary Material

Additional File 1 Supplementary material for gene ambiguity for mining MEDLINE Click here for file

## Figures and Tables

**Table 1:** Information of abbreviation data set

Abbreviation	Sense #	Sense	# of retrieved articles	Sense Distribution
BPD	BPD1	borderline personality disorder	1584	32%
	BPD2	bronchopulmonary dysplasia	2335	47%
	BPD3	biparietal diameter	1032	21%

BSA	BSA1	bovine serum albumin	13352	89%
	BSA2	body surface area	5815	11%

PCA	PCA1	posterior cerebral artery	1165	67%
	PCA2	posterior communicating artery	584	33%

RSV	RSV1	respiratory syncytial virus	5295	60%
	RSV2	rous sarcoma virus	3520	40%

**Table 2:** Results for *BSA *data set. Annotation of the table: Dist: Distribution of senses; S. Size: sample size; Err. Rate: Error Rate; SE: Standard Error of error rates; CV: cross-validation;

**BSA**		**5-fold CV**		**10-fold CV**	
Dist	S. Size	Err. Rate	SE	Err. Rate	SE

(0.5, 0.5)	20	21.83%	10.05%	19.67%	9.04%
	40	11.17%	5.33%	11.08%	5.05%
	80	5.08%	2.60%	5.04%	2.44%
	120	3.11%	1.72%	2.61%	1.48%

(0.6, 0.4)	20	23.50%	10.21%	21.00%	9.21%
	40	12.67%	5.75%	12.08%	5.34%
	80	5.75%	2.82%	5.00%	2.48%
	120	3.58%	1.85%	3.28%	1.67%

**(0.7, 0.3)**	20	24.33%	10.59%	23.00%	9.74%
	40	14.67%	6.11%	12.75%	5.39%
	80	7.17%	3.16%	6.67%	2.87%
	120	4.86%	2.17%	4.00%	1.85%

(0.8, 0.2)	20	19.33%	9.82%	19.33%	9.27%
	40	15.33%	6.31%	14.08%	5.72%
	80	9.13%	3.58%	8.00%	3.16%
	120	5.22%	2.23%	4.53%	1.96%

(0.9, 0.1)	20	10.17%	7.55%	10.00%	7.07%
	40	10.17%	5.33%	10.00%	4.99%
	80	8.00%	3.38%	7.71%	3.13%
	120	6.42%	2.48%	6.03%	2.26%

**Table 3:** Results for *PCA *data set. Annotation of the table: Dist: Distribution of senses; S. Size: sample size; Err. Rate: Error Rate; SE: Standard Error of error rates; CV: cross-validation;

**PCA**		**5-fold CV**		**10-fold CV**	
Dist	S. Size	Err. Rate	SE	Err. Rate	SE

(0.5, 0.5)	20	43.00%	12.14%	41.00%	11.25%
	40	34.58%	8.21%	34.33%	7.68%
	80	37.17%	5.44%	29.46%	5.14%
	120	28.53%	4.45%	31.47%	4.13%

(0.6, 0.4)	20	37.83%	11.62%	38.50%	11.04%
	40	36.42%	8.12%	35.92%	7.37%
	80	25.54%	5.41%	24.88%	5.06%
	120	28.22%	4.25%	29.25%	3.96%

(0.7, 0.3)	20	33.67%	11.48%	33.50%	10.90%
	40	33.08%	8.06%	33.08%	7.62%
	80	29.67%	5.38%	24.29%	4.98%
	120	26.83%	4.36%	27.83%	4.11%

(0.8, 0.2)	20	23.67%	10.48%	24.50%	9.99%
	40	21.83%	7.01%	20.58%	6.61%
	80	28.00%	5.09%	19.25%	4.61%
	120	22.92%	3.97%	25.03%	3.65%

(0.9, 0.1)	20	12.33%	8.14%	12.00%	7.59%
	40	10.92%	5.48%	11.08%	5.20%
	80	14.04%	4.10%	12.50%	3.79%
	120	10.42%	3.11%	11.14%	2.98%

**(0.67,0.33)**	20	38.33%	11.89%	36.33%	10.96%
	40	30.17%	7.93%	29.50%	7.46%
	80	28.25%	5.43%	24.83%	5.00%
	120	29.47%	4.50%	35.33%	4.15%

**Table 4:** Results for *RSV *data set. Annotation of the table: Dist: Distribution of senses; S. Size: sample size; Err. Rate: Error Rate; SE: Standard Error of error rates; CV: cross-validation;

**BSA**		**5-fold CV**		**10-fold CV**	
Dist	S. Size	Err. Rate	SE	Err. Rate	SE

(0.5, 0.5)	20	26.50%	10.52%	27.00%	9.72%
	40	18.83%	6.83%	17.83%	6.29%
	80	12.79%	4.09%	12.17%	3.78%
	120	10.58%	3.10%	10.69%	2.93%

**(0.6, 0.4)**	20	27.83%	10.78%	27.67%	10.09%
	40	20.25%	7.00%	19.50%	6.52%
	80	13.67%	4.25%	12.83%	3.91%
	120	11.53%	3.20%	10.39%	2.90%

(0.7, 0.3)	20	27.33%	10.84%	26.33%	10.18%
	40	19.00%	6.81%	17.83%	6.23%
	80	13.96%	4.27%	13.08%	3.91%
	120	11.56%	3.23%	10.86%	2.97%

(0.8, 0.2)	20	21.50%	10.20%	19.50%	9.20%
	40	17.08%	6.60%	16.75%	6.17%
	80	14.00%	4.29%	13.29%	3.96%
	120	11.69%	3.26%	10.75%	2.96%

(0.9, 0.1)	20	11.00%	7.77%	10.67%	7.25%
	40	10.58%	5.42%	10.33%	5.05%
	80	9.54%	3.66%	9.33%	3.41%
	120	8.67%	2.86%	8.36%	2.65%

**Table 5:** Results for *BPD *data set. Annotation of the table: Dist: Distribution of senses; S. Size: sample size; Err. Rate: Error Rate; SE: Standard Error of error rates; CV: cross-validation;

**BPD**		**5-fold CV**						**10-fold CV**					
		mc-svm		one-vs-rest		one-vs-one		mc-svm		one-vs-rest		one-vs-one	
		
Dist.	S. size	Err. Rate	SE	Err. Rate	SE	Err. Rate	SE	Err. Rate	SE	Err. Rate	SE	Err. Rate	SE

(0.33, 0.33, 0.33)	30	26.56%	8.77%	25.78%	8.68%	29.22%	9.06%	23.89%	8.05%	23.44%	7.96%	26.22%	8.31%
	60	13.89%	4.89%	13.39%	4.83%	15.83%	5.18%	11.89%	4.30%	11.56%	4.23%	13.78%	4.62%
	120	7.67%	2.66%	7.08%	2.55%	8.44%	2.80%	7.00%	2.40%	6.39%	2.31%	8.06%	2.58%
	180	6.06%	1.96%	5.70%	1.90%	6.69%	2.05%	5.70%	1.79%	5.20%	1.72%	6.24%	1.88%

(0.6, 0.2, 0.2)	30	26.33%	8.91%	25.33%	8.75%	26.89%	8.97%	24.67%	8.21%	23.89%	8.06%	25.44%	8.30%
	60	16.28%	5.27%	15.56%	5.16%	17.67%	5.44%	15.33%	4.85%	14.00%	4.65%	16.33%	4.97%
	120	10.11%	3.05%	9.22%	2.93%	10.50%	3.10%	9.36%	2.78%	8.50%	2.66%	10.00%	2.87%
	180	7.72%	2.21%	6.89%	2.09%	8.09%	2.26%	6.93%	1.98%	6.37%	1.91%	7.41%	2.04%

(0.8, 0.1, 0.1)	30	18.11%	7.82%	18.11%	7.83%	19.00%	7.99%	18.33%	7.41%	18.22%	7.40%	19.00%	7.53%
	60	14.78%	5.10%	14.28%	5.03%	15.39%	5.18%	14.67%	4.79%	13.83%	4.67%	14.78%	4.81%
	120	9.31%	2.95%	8.69%	2.85%	9.50%	2.98%	8.56%	2.67%	8.06%	2.59%	8.75%	2.70%
	180	6.87%	2.09%	6.59%	2.05%	7.17%	2.14%	6.35%	1.91%	5.87%	1.84%	6.61%	1.94%

**(0.32, 0.47, 0.21)**	30	24.22%	8.58%	23.33%	8.44%	26.67%	8.84%	23.00%	7.93%	21.67%	7.71%	25.22%	8.17%
	60	15.89%	5.21%	14.89%	5.08%	16.83%	5.34%	14.11%	4.66%	13.33%	4.55%	15.39%	4.80%
	120	9.19%	2.91%	7.92%	2.71%	10.25%	3.07%	8.36%	2.64%	7.53%	2.50%	9.50%	2.80%
	180	6.07%	1.95%	5.48%	1.85%	6.78%	2.07%	5.39%	1.73%	4.61%	1.61%	6.07%	1.85%
