# Supplementary material for: Machine learning and word sense disambiguation in the biomedical domain: design and evaluation issues
Source: BMC Bioinformatics. 2006 Jul 5;7:334. doi: 10.1186/1471-2105-7-334 (PMC1550263; doi:10.1186/1471-2105-7-334)
Supplement: Additional File 1 — Supplementary material for gene ambiguity for mining MEDLINE [file 1471-2105-7-334-S1.doc]

## Additional file 1: supplementary material

## Study of gene name ambiguity for mining MEDLINE

To demonstrate the extent of the ambiguity problem in MEDLINE we searched MEDLINE abstracts to determine how many abstracts contained gene symbols that were ambiguous with general English words or biomedical terms.

We formed a mouse gene symbol list by retrieving all gene symbol/name/synonyms from Entrez Gene, the gene-specific database at the National Center for Biotechnology Information (NCBI), for the mouse species. Then we compared this gene symbol list with a general English word list (Webster’s 2^nd^ international dictionary) and with the UMLS term list (from UMLS Metathesaurus 2005AA, removing all bio-molecular entities with semantic types “Gene or Genome”, “Biologically Active Substance”, “Amino Acid”, “Peptide or Protein”, “Enzyme”, “Immunologic Factor and Receptor”) via case-insensitive exact string match. Two ambiguous gene symbol lists were formed as the result of the comparisons: a gene-English list (containing gene symbols ambiguous with general English words) and a gene-UMLS list (containing gene symbols ambiguous with biomedical terms). We also formed a pool of MEDLINE abstracts by collecting all abstracts that were related to mouse genes using *gene2pubmed* file from Entrez Gene (downloaded on 1/2006), which led to 82, 922 abstracts in the pool. We performed a case-insensitive search on each abstract in the pool to determine the number of abstracts that contained at least one word in each of the above two lists respectively, so that we could determine the percent of abstracts that contained a word that was ambiguous with an English word or with a UMLS term respectively. We repeated the same procedure for the fly and yeast organisms as well. Results showed that for the mouse organism alone, 99.7% (82694/82922) of the abstracts were affected by an ambiguity between a gene symbol and a general English word, and 99.8% (82736/82922) were affected by an ambiguity between a gene symbol and a UMLS term. For the fly organism, both numbers were also over 99%, while the number was much less for the yeast organism: 4.6% and 3.1% respectively.

However there is a concern that a very limited set of words may have accounted for the vast majority of ambiguity. Therefore, for each ambiguous word, we calculated its frequency, which is defined as the ratio between the number of abstracts containing the word and the total number of abstracts in the pool. For example, the word “brown” occurred in 399 abstracts therefore had a frequency of 399 / 82922 = 0.48%. Table 1 shows some sample terms with different frequencies.

For each threshold, we removed ambiguous words with frequencies higher than that threshold and re-calculated the percentage of abstracts that contained the remaining ambiguous words. Meanwhile, we also recorded the percentage of ambiguous words that were removed from the ambiguous word-list for different thresholds. For example, the gene-UMLS list of the mouse organism has total of 2064 ambiguous words and the frequency of each word was calculated. When we set the threshold to 10%, there were 20 ambiguous words with frequencies higher than 10% and they were removed from the gene-UMLS list. By searching the abstracts pool, there were still 89.8% of abstracts containing at least one of the remaining 2044 ambiguous words. We recorded the percentage of words removed as 20/2064=0.97%. As shown in Figure 1, we removed words with frequencies higher than 10%, 1%, 0.1% and 0.05% from the two lists of the mouse organism. Results showed that the percentages of abstracts containing the remaining ambiguous words were 80.9%, 46.2%, 13.5% and 7.2% respectively for gene-English ambiguity, and 89.8%, 68.6%, 24.0% and 13.4% respectively for gene-UMLS ambiguity. Figure 2 shows the corresponding percentages of ambiguous words that were removed from the lists at different thresholds (10%, 1%, 0.1%, 0.05%), which were 0.8%(8/1065), 4.8%(51/1065), 20.3%(216/1065), 30%(319/1065) for gene-English ambiguity and 1.0%(20/2064), 3.8%(79/2064), 21.2%(437/2064) and 30.8%(636/2064) for gene-UMLS ambiguity. As those figures showed, in order to reduce the percent of abstracts with ambiguity from gene-English and gene-UMLS to a relative low level (7.2% and 13.4% respectively), ambiguous words with frequencies higher than 0.05% would have to be removed, which covered 30.0% and 30.8% of all the ambiguous words in the two lists respectively. The same study was also done for the Fly organism and showed similar results, but with slightly higher ambiguity rates. This study shows that the ambiguity among gene symbols, English words and other biomedical terms is extensive and the distribution of ambiguity is very sparse. Therefore word sense disambiguation is critical for the text mining and retrieval tasks in the biomedical domain.

| Type of ambiguity | Total number ambiguous words | Words with frequency > 10% | Words with frequency > 1% | Words with frequency > 0.1% | Words with frequency > 0.05% |
| --- | --- | --- | --- | --- | --- |
| Gene-Eng | 1065 | receptor, brain | fat,  camp | cat,  tag | ship,  trail |
| Gene-UMLS | 2064 | can (chronic allograft nephropathy),  mice | skin,  mg (milligram or magnesium) | nodal,  mcp (metoclopramide) | hip,  mottled |

Table 1: sample ambiguous words with different frequencies for mouse organism. Some UMLS abbreviations were showed with their full terms in the parenthesis.

Figure 1: After removing terms with frequencies higher than a set of thresholds: 10%, 1%, 0.10% and 0.05%, the percentages of articles containing remaining ambiguous terms were as following: 80.9%, 46.2%, 13.5% and 7.2% for gene-English ambiguity, and 89.8%, 68.6%, 24.0% and 13.4% for gene-UMLS ambiguity.

Figure 2: After removing terms with frequencies higher than a set of thresholds: 10%, 1%, 0.10% and 0.05%, following percentages of words were removed from two lists: 0.8%, 4.8%, 20.3%, 30% for gene-English list and 1.0%, 3.8%, 21.2% and 30.8% for gene-UMLS list.
